# Finding of a two-headed green turtle embryo during nest monitoring in Baa Atoll, Maldives

**DOI:** 10.4102/ojvr.v88i1.1940

**Published:** 2021-08-24

**Authors:** Stephanie Köhnk, Rosie Brown, Amelia Liddell

**Affiliations:** 1 Olive Ridley Project, Bramhall, Stockport, Cheshire, United Kingdom; 2 Morphology Lab, Center of Natural History, University of Hamburg, Hamburg, Germany; 3 Marine Turtle Rescue Centre, Coco Palm Dhuni Kolhu, Maldives

**Keywords:** case report, *Chelonia mydas;* congenital malformation, polycephaly, sea turtle, nesting, Indian Ocean

## Abstract

Green sea turtles are one of the two species of marine turtles known to nest in the Maldives. The prevalent time of nesting seems to be inconsistent throughout the island nation. In this study, sea turtle nesting activity was monitored on the island of Coco Palm Dhuni Kolhu in Baa Atoll over a period of 12 months. A total of 13 nests were confirmed with a median hatching success rate of 89.58% as ascertained by nest excavation. In one of the nests, a severely deformed hatchling with polycephaly, an opening in the neck area and a lordotic spine was found, and we investigated in detail with radiographic images and a necropsy. Our findings support the importance of consistent nesting activity and nest monitoring efforts in the country as a basis for conservation efforts.

## Introduction

Congenital malformations in wildlife are only reported sporadically, but they have been found in fishes (Dethelfsen, Von Westerhagen & Cameron 1996; Hevia-Homazábal, Pasten-Marambio & Vega 2011), amphibians (Blaustein & Johnson 2003; Henle et al. 2012) and reptiles (Frye 1991; Sant’Anna et al. 2013), birds (Ohlendorf et al. 1986; Pourlis 2011) and mammals (Rojas-Lleonart, Silveira-Prade & Sotero-Delgado 2011; Stills & Bullock 1981). In sea turtles, various case reports from different populations all over the world are known (see, e.g., Bárcenas-Ibarra et al. 2015; Caldwell 1959; Carswell & Lewis 2002; Craven et al. 2019; Dodd 1988; Drennen 1990; Eckert et al. 2012; Ehrhart & Witherington 1987; Fowler 1979; Gularte 2000; Hughes, Bass & Mentis 1967; Kaska & Downie 1999; Reichart 1993; Rhodin, Pritchard & Mittermeier 1984).

Malformations can range in severity from benign scute abnormalities to detrimental conditions such as ectromelia or deformities of the spine (Bárcenas-Ibarra et al. 2015; Dodd 1988; Miller 1982; Rhodin et al. 1984). Early reports of developmental abnormalities date back to the 19th century, when Agassiz (1857) described cases of scute irregularities. This is the most common abnormality observed in sea turtles (see Dodd 1988 for a summary), which is not detrimental on its own because adult turtles with abnormal scute patterns are spotted regularly (Bentley et al. 2021; Brongersma 1968; Kobayashi et al. 2017a; Türkozan, Illgaz & Sak 2001).

More serious abnormalities can include twinning, deformities of the eyes, the jaw or the entire body and carapace, a reduction or absence of limbs, di- or polycephaly as well as leucism and albinism (Bárcenas-Ibarra et al. 2015; Caldwell 1959; Carswell & Lewis 2002; Dodd 1988; Drennen 1990; Eckert et al. 2012; Ehrhart & Witherington 1987; Fowler 1979; Gularte 2000; Hughes et al. 1967; Ingle et al. 2021; Kaska & Downie 1999; Reichart 1993; Rhodin et al. 1984), which have been documented in loggerheads, hawksbills, green turtles, leatherbacks and olive ridleys. The prevalence of congenital malformations can differ between sea turtle populations but overview studies are currently still rare (see e.g. Bárcenas-Ibarra et al. 2015; Calderón Peña & Azanza Ricardo 2021; Craven et al. 2019; Fowler 1979; Kaska, Downie & Furness 2000; Peters, Verhoeven & Strijbosch 1994).

Previous case reports from the Indian Ocean include a study from Diamond (1976) on hawksbill turtles in the Seychelles in which the author found two cases of deformed hatchlings. These showed a transverse medial fold across the body and, though viable at the time of hatching, did not survive past 5 and 9 weeks, respectively. Other studies described instances of twinning in Sri Lanka (Deraniyagala 1930, 1932; Hewavisenthi 1989), Thailand (Junchompoo, Penpian & Tarkoolrangsi 2013), Malaysia (Chan 1985) and Mozambique (Louro & Pereira 2009), as well as supernumerary suprapygal bones in loggerheads in Sri Lanka (Deraniyagala 1939) and kyphosis in green turtles in Indonesia (Rhodin et al. 1984).

In the Maldives, an island nation in the northern Indian Ocean, two species of sea turtles are known to nest regularly: green and hawksbill turtles (Ahmed et al. 2020; Ali & Shimal 2016; Hudgins et al. 2017). Nesting is spread over the entire length of the country and occurs all year round with hatching success rates between 33.7% and 96.8% (Hudgins et al. 2017). No general information or overview study on unhatched eggs, embryo or hatchling malformations is available for the Maldives at the time of this publication.

Herein, we report on sea turtle nesting activity in 2020 in Coco Palm Dhuni Kolhu (CPDK), Baa Atoll, Maldives, including the case of an embryo with congenital malformations.

## Materials and Methods

Sea turtle nesting activity was recorded on CPDK, Baa Atoll, Maldives throughout the year (in agreement with the guidelines of the Environmental Protection Agency of the Maldives, research permits EPA/2018/PSR-T03 and EPA/2020/PSR-T06). Nesting attempts and true nests were recorded, and nests were monitored throughout the incubation period until hatching occurred. Nests were excavated, and the nests’ contents were analysed at least 2 days after the emergence of the first hatchling, to allow for natural hatching of any stragglers. Variation in excavation times resulted from coronavirus disease 2019 (COVID-19)related safety measures and organisational restrictions. Any unhatched eggs were opened by hand, pinching the eggshell between two fingers and pulling apart to reveal the egg contents, which were then classified according to the criteria specified in Table 1 and reburied at the site of the nest.

**TABLE 1:** Categorisation criteria for unhatched eggs examined during nest excavations.

Category	Criteria
Undeveloped	No evidence of embryo visible, only yolk
Stage 1	Evidence of embryo < 1 cm, not pigmented
Stage 2	Embryo < 3 cm, visibly pigmented
Stage 3	Embryo 3 cm – 4 cm, large yolk attached
Stage 4	Embryo > 4 cm, small yolk sac, embryo fills biggest portion of the egg
Pipped	Hatchling has pierced through the shell but not fully emerged

Source: Adapted from Chacón, D., Dick, B., Harrison, E., Sarti, L., & Solano, M., 2008, ‘Manual sobre técnicas de manejo y conservación de las tortugas marinas en las playas de anidación de Centroamérica’, *Secretaría Pro Tempore de la Convención Interamericana para la Prioteccióny Conservación de las Tortugas Marinas (CIT)*, San José, Costa Rica.

The single malformed embryo was found in one of the nests and was further studied to determine the extent of the malformation as best as possible. The embryo was intact within its egg, in a normal foetal position (back flippers curled in a cranial direction, following the curve of the egg; front flippers curled in a medial direction, coming to rest in contact with and cranial to the back flippers). It had reached a more advanced stage of development (stage 4) but had not survived to the point of pipping. Radiographic images were obtained with a Veterinary X-rays High Frequency Diagnostic Unit machine in four standard views; dorsoventral, lateral (right and left), craniocaudal and ventrodorsal, followed by a standard necropsy (Work 2000) to characterise any duplication of internal organs and the points of fusion. No further tissue samples were taken for histological or genetic evaluation.

### Ethical considerations

This article followed all ethical standards for research without direct contact with human or animal subjects.

## Results

### Nesting activity

In 2020, we recorded 13 false crawls (nesting attempts) and 13 confirmed nests on the island CPDK between April and October. All false crawls, failed nest attempts and nests were identified as green turtle (*Chelonia mydas*) activities with confirmed nests restricted to June to September (Table 2).

**TABLE 2:** Recorded false crawl and confirmed nesting events on Coco Palm Dhuni Kolhu in 2020.

No.	Incidence	Date	Relocated (Yes/No)	Hatching date	Excavation date	Number of eggs	Number of hatchlings	Unhatched eggs(UD-1-2-3-4-P)	Hatching success rate (%)	Depth in cm
1	FC	14.04.20	-	-	-	-	-	-	-	-
2	FC	17.04.20	-	-	-	-	-	-	-	-
3	FC	28.04.20	-	-	-	-	-	-	-	-
4	FC	06.05.20	-	-	-	-	-	-	-	-
5	FC	09.05.20	-	-	-	-	-	-	-	-
6	Nest	02.06.20	N	NA	28.07.20	30	22	8-0-0-0-0-0	73.33	NA
7	Nest	21.07.20	N	20.09.20	21.09.20	109	102	7-0-0-0-0-0	93.58	NA
8	FC	26.07.20	-	-	-	-	-	-	-	-
9	Nest	28.07.20	Y	25.09.20	27.09.20	142	57	79-0-2-0-2-0	40.14	64–75
10	FC	01.08.20	-	-	-	-	-	-	-	-
11	Nest	01.08.20	Y	01.10.20	03.10.20	104	66	32-1-2-1-1-1	63.46	57–77
12	FC	08.08.20	-	-	-	-	-	-	-	-
13	Nest	08.08.20	Y	NA	03.10.20	112	103	4-0-0-1-2-2	92.86	53–84
14	Nest	10.08.20	N	09.10.20	15.10.20	142	126	10-0-3-1-1-0	88.73	57–70
15	Nest	20.08.20	N	21.10.20	23.10.20	122	82	33-0-2-5-0-0	67.21	59–81
16	Nest	29.08.20	N	27.10.20	28.10.20	114	105	7-0-1-1-0-0	92.11	48–74
17	FC	31.08.20	-	-	-	-	-	-	-	-
18	Nest	01.09.20	N	30.10.20	02.11.20	94	85	7-0-0-2-0-0	90.43	59–78
19	Nest	09.09.20	Y	06.11.20	09.11.20	98	82	8-5-0-1-1-0	83.67	58–80
20	Nest	09.09.20	N	05.11.20	09.11.20	141	124	4-0-1-1-1-0	93.62	64–85
21	FC	20.09.20	-	-	-	-	-	-	-	-
22	FC	20.09.20	-	-	-	-	-	-	-	-
23	FC	21.09.20	-	-	-	-	-	-	-	-
24	Nest	21.09.20	N	21.11.20	24.11.20	118	107	4-0-2-0-0-0	90.68	55–82
25	FC	23.09.20	-	-	-	-	-	-	-	-
26	FC	02.10.20	-	-	-	-	-	-	-	-

Classification of undeveloped eggs is recorded as follows: UD – 1, Stage 1 embryo; 2, Stage 2 embryo; 3, Stage 3 embryo; 4, Stage 4 embryo; P, pipped.

FC, false crawl; UD, undeveloped.

Egg counts ranged between 30 and 142 eggs in confirmed nests (median = 113; *N* = 12) and hatching success rate was 40.14% – 93.62% (median success rate of 89.58%; *N* = 12, s.d. = 16.57%) after an average of 59 days of incubation. The unhatched eggs in the 12 excavated nests were mostly completely undeveloped, showing no signs of embryo development (for details, also see Table 2).

**FIGURE 1:**
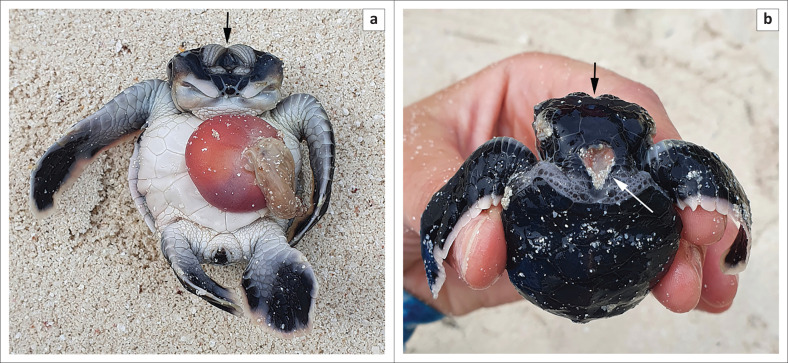
Hatchling with polycephaly after it was removed from the egg. (a) Ventral view, prominent yolk sac present. Arrow indicating the fused eyelid. (b) Anterior-dorsal view. Point of fusion on the head indicated by black arrow, dorso-cervical opening indicated by the white arrow.

In a nest laid on 10 August 2020, that was estimated to have hatched on 09 October 2020 and excavated on 15 October 2020, a malformed embryo was found (Figure 1).

### The two-headed specimen

**FIGURE 2:**
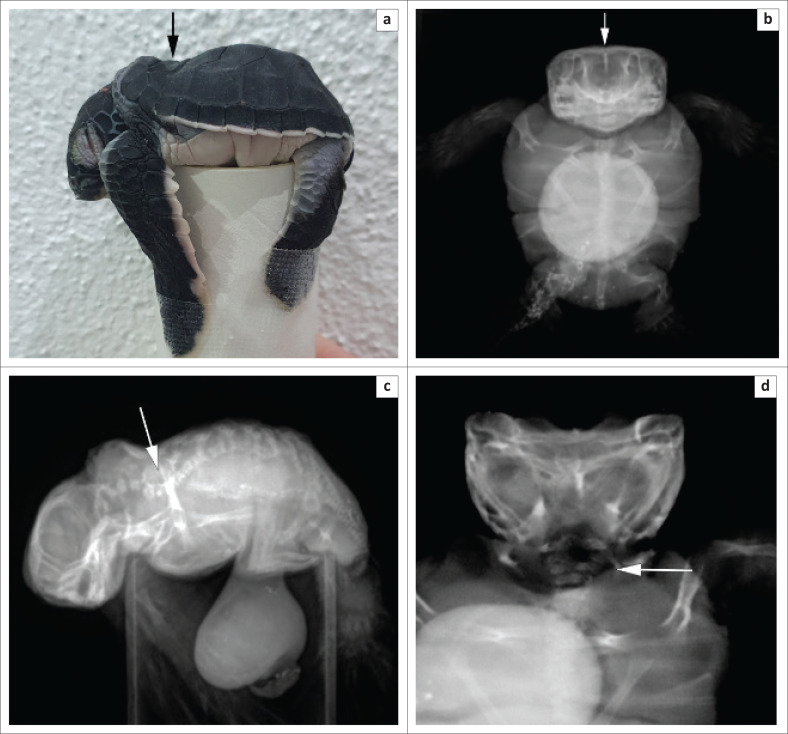
Radiographic images of the malformed hatchling. (a) Positioning of the hatchling for lateral image acquisition – spinal deformation clearly visible (arrow). (b) Ventral view – yolk sac visible and fusion of the two skulls indicated by arrow. (c) Lateral view – lordotic region of the spin indicated by arrow. (d) Dorsal view – point of fusion of the two necks indicated by arrow.

This embryo represents a case of polycephaly, with the heads incompletely separated down the midline (Figure 1a). Externally, the heads fused at the supraorbital scale and caudal to the rhamphotheca (Figure 1). Both heads shared a medial canthus of the eyelid, but each eye had a distinct globe. Additionally, external deformations included the presence of an opening in the dorsal cervical region (Figure 1b) and a lordotic region in the spine and carapace (Figure 2a and c).

**FIGURE 3:**
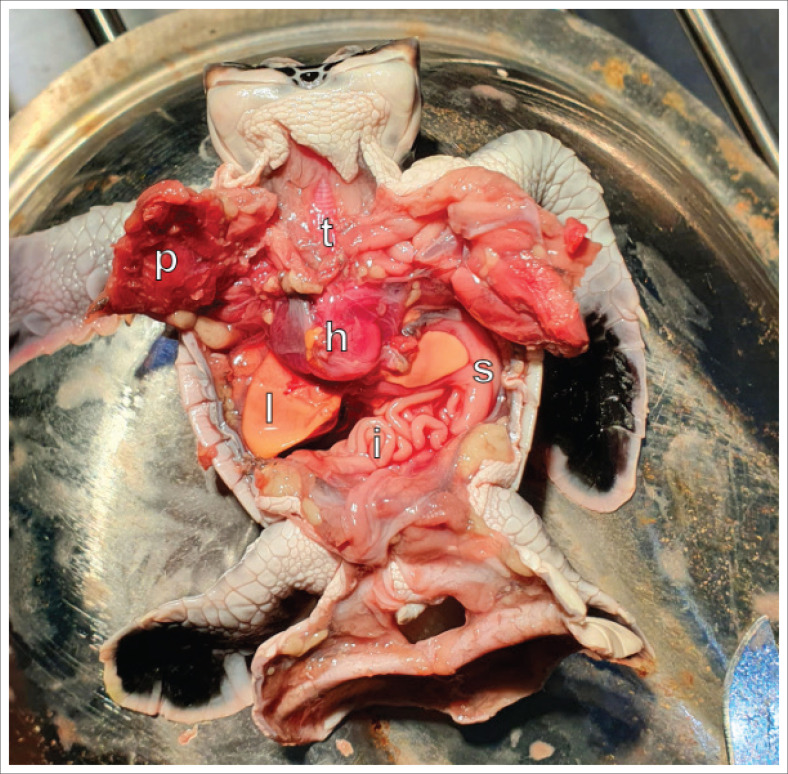
Necropsy image of the hatchling with plastron removed and pectoral muscles reflected cranially showing normal organ development without duplications below the neck. All organs present and visually assessed as normal in appearance, size, colour and texture. *h* = heart, *i* = intestine, *l* = liver, *p* = pectoral muscle, *s* = stomach, *t* = trachea.

Radiographic images indicated the fusion of the skulls at approximately the level of the post orbital bones (Figure 2b). Each head had its own cervical vertebrae down to approximately the level of cervical vertebrae 3 and 4, where they fused to continue down into a relatively normal single embryo body (Figure 2d), apart from the lordosis at approximately the level of the first and second thoracic vertebrae (Figure 2c). Because of the presence of a significant yolk sac and the small size of the embryo (straight carapace length = 38.4 mm, straight carapace width = 35.4 mm and snout vent length left head = 54.6 mm), the coelomic contents were indistinct on the radiographic images, but the axial and appendicular skeletons were more easily distinguishable because of the higher density of bone – and therefore greater absorption of X-rays – than soft tissue (fat, muscle and viscera). All four flippers were anatomically normal in all respects, as confirmed during the following necropsy. The necropsy demonstrated that the embryo had a normal coelomic cavity, with all organs present and with no duplication, aplasia or abnormalities (Figure 3). All organs had a normal appearance, size, colour and texture based on visual assessment. At the approximate level of the thoracic inlet, the oesophagus and trachea split to serve each separate head, which each had a distinct and normally formed oral cavity, nares and glottis. The cause for the opening on the dorsal surface of the neck was unclear as the anatomical features were hard to distinguish during dissection. The fusion or lack, thereof, of the brain was not assessed because of the lack of appropriate tools to adequately expose the brain without destroying it in the process.

## Discussion

### Nesting and hatching success on Coco Palm Dhuni Kolhu

In general, nests recorded in 2020 on CPDK showed a relatively high median hatching success rate of almost 90%. As embryonic development can reflect the environmental conditions during incubation (see e.g. Bailey et al. 2018; Booth 2017; Kaska & Downie 1999; Kobayashi et al. 2017b; Miller 1985; Packard 1991; Rafferty & Reina 2012), the high hatching success rate in this case can indicate good incubation conditions on the island. Reptile nests are highly sensitive to specific temperatures and humidity (Deeming 2004; Fuentes, Limpus & Hamann 2011; Segura & Cajade 2010). Successful nests again point towards great incubation conditions for sea turtle nests on CPDK. Comparable hatching rates have been documented in the entire country (Hudgins et al. 2017), showing that beach conditions in the Maldives are currently well-suited for sea turtle nests, providing these nests are left to develop undisturbed. Previous studies worldwide showed a reduction in hatching success rates in nests that were relocated (Ahles & Milton 2016; Eckert & Eckert 1990; Hudgins et al. 2017; Pintus et al. 2006).

In this study, four nests were relocated because of closeness to the high water mark. This was done within 12 h after oviposition in three cases, as careful movement of eggs is less likely to be detrimental within that time frame (Parmenter 1980). The exception was one nest (Table 2, number 19), which was unexpectedly washed out at a late point in incubation during a bad weather event. The decision was made to relocate the nest at the later point in development, because of the direct risk of losing the nest.

The combination of a large number of false crawls and no confirmed nests in the first two months of activity may point towards a variability in fine-scale natal homing, which is known from other green turtle populations (Nishizawa et al. 2011; Shamblin et al. 2020). Turtles could be nesting on neighbouring islands within a certain distance, and additional studies are needed to determine the extent of natal homing ranges in green turtles in the Maldives.

### Congenital malformations

Apart from benign scute abnormalities, the most common aberrations from a normal developmental pattern include changes to the limbs, followed by changes to the head (Craven et al. 2019), the latter being the case presented here.

According to Miller (1985), skull development starts as early as 6–11 days after incubation, pointing towards the development of the malformation of the presented embryo in the earliest stages of incubation. Any errors occurring in the first half of development, within the first 30 days, respectively, can have both congenital and environmental factors as an origin (Bárcenas-Ibarra et al. 2015; Dodd 1988; Drennen 1990) and often prove to be fatal. This leaves the exact cause of the observed abnormalities to speculate in many cases, as in the one presented here. The malformed embryo found on CPDK died inside the egg, as would be expected as more serious deformities, such as polycephaly in combination with a cervical opening, are often fatal prior to hatching or even pipping (Miller 1985). The opening in the neck likely represents an incomplete fusion of the spinal canal or an associated defect of the neural crest. Together with the presence of two heads, this might be caused by terminal bifurcation of the notochord during neurulation, as documented in other vertebrates (Spemann & Mangold 1924; Twigg & Wilikie 2015), or because of the incomplete separation of early embryos (see Wu et al., 2002 for a review).

Similar to general hatching success, environmental conditions can influence the chance of abnormalities in development with temperature and humidity playing a prominent role. According to McGehee (1979), incubation at overall lower temperatures (< 30 °C) can increase the chance of hatchling deformities. Most likely though, it is not a low temperature alone that triggers aberrant development but a combination with other environmental or hereditary factors (Miller 1982), similar to associated causes reported in snakes (Wallach 2007). As the Maldives have comparably stable high temperatures year-round, the influence of low temperature on the development of the hatchling is considered unlikely, but sand temperature measurements should be taken to confirm this assumption.

Additionally, various components such as polycyclic aromatic hydrocarbons or mercury have been identified as possible agents changing deoxyribonucleic acid (DNA) methylation patterns and thus triggering abnormal development (Bárcenas-Ibarra et al. 2015; see Martín-del-Campo, Sifuentes-Romero & Garcías-Gasca 2019; Martín-del-Campo et al. 2021 for a summary). The prevalence of such components in sea turtle eggs in the Maldives is currently unknown.

## Conclusion

As the Maldives is a rather widespread country with many small individual islands, systematic evaluation of sea turtle nesting activity is still problematic. For accurate estimates of natal homing ranges of individual females and thus possible population management units within the nesting green turtle population of the Maldives, consistent monitoring efforts need to be extended. Inclusion of islands with known nesting activity as well as their neighbouring islands will be necessary for the quantification of the resident nesting population and its internal structure. Monitoring and nest excavation efforts are also essential for evaluating the overall frequency of congenital malformations, which are known from sea turtle populations worldwide but seem to appear at different frequencies within different populations. Consistent monitoring is needed as a basis to investigate environmental factors, causes and triggers for such malformations, which is especially relevant in the Maldives in the light of continued island and beach development all over the country.
